# Microfluidic Bi-Layer Platform to Study Functional Interaction between Co-Cultured Neural Networks with Unidirectional Synaptic Connectivity

**DOI:** 10.3390/mi14040835

**Published:** 2023-04-11

**Authors:** Yana Pigareva, Arseniy Gladkov, Vladimir Kolpakov, Anton Bukatin, Sergei Li, Victor B. Kazantsev, Irina Mukhina, Alexey Pimashkin

**Affiliations:** 1 Neurotechnology Department, Lobachevsky State University of Nizhny Novgorod, Nizhny Novgorod 603950, Russia; 2 Central Research Laboratory, Cell Technology Department, Privolzhsky Research Medical University, Nizhny Novgorod 603005, Russia; 3 Department of Nanobiotechnology, Alferov Saint-Petersburg National Research Academic University of the Russian Academy of Sciences, Saint Petersburg 194021, Russia; 4 Institute for Analytical Instrumentation of the RAS, Saint Petersburg 198095, Russia

**Keywords:** microfluidics, neuronal tissue engineering, organ-on-a-chip, microfluidic cell culture, drug discovery, coculture techniques, microchannels, microelectrode arrays

## Abstract

The complex synaptic connectivity architecture of neuronal networks underlies cognition and brain function. However, studying the spiking activity propagation and processing in heterogeneous networks in vivo poses significant challenges. In this study, we present a novel two-layer PDMS chip that facilitates the culturing and examination of the functional interaction of two interconnected neural networks. We utilized cultures of hippocampal neurons grown in a two-chamber microfluidic chip combined with a microelectrode array. The asymmetric configuration of the microchannels between the chambers ensured the growth of axons predominantly in one direction from the Source chamber to the Target chamber, forming two neuronal networks with unidirectional synaptic connectivity. We showed that the local application of tetrodotoxin (TTX) to the Source network did not alter the spiking rate in the Target network. The results indicate that stable network activity in the Target network was maintained for at least 1–3 h after TTX application, demonstrating the feasibility of local chemical activity modulation and the influence of electrical activity from one network on the other. Additionally, suppression of synaptic activity in the Source network by the application of CPP and CNQX reorganized spatio-temporal characteristics of spontaneous and stimulus-evoked spiking activity in the Target network. The proposed methodology and results provide a more in-depth examination of the network-level functional interaction between neural circuits with heterogeneous synaptic connectivity.

## 1. Introduction

The synaptic connectivity architecture of neuronal networks plays a crucial role in cognition and brain function. Both in vivo and in silico studies have shown that information processing occurs in node-like and modular neuronal circuit topologies [1]. Features of spiking activity generation and propagation on the network level are important to understand information processing mechanisms in the brain. However, studying the complex 3D synaptic architecture and its impact on electrophysiological activity propagation and processing in living organisms or slices remains a significant challenge. To address this challenge, recent advancements in microfluidic techniques and cell culturing methods have enabled the separation of neuronal cell bodies and axons, and directed axonal growth to form network structures that are similar to those in vivo. This has been achieved through the use of microfluidic chips composed of chambers and microchannels that guide axons and create unidirectionally connected modular neural networks [2,3,4,5,6,7,8,9]. Several studies have modeled two interconnected networks of neurons from different brain regions, such as the thalamo-cortical system [10], cortical-striatal systems [11,12,13], various regions of the hippocampus [14,15,16,17], and the cortex coupled with the hippocampus [18,19]. A three-chamber chip was used to study the interactions of cells from different brain regions in vitro, including the prefrontal cortex, hippocampus, and amygdala; as well as its application to the pharmacological manipulation of individual networks and their effects on interconnected regions [20]. Furthermore, a brain-regions-on-a-chip model has been used to compare the interactions between the cortex, hippocampus, and thalamus [21].

The functional mechanisms of neuronal network interactions in brain-on-a-chip models are studied using imaging techniques and multisite extracellular electrophysiology with microelectrode arrays (MEAs). MEAs are a powerful tool for long-term monitoring of network activity and have been used in numerous studies of memory [22,23,24], plasticity [25,26,27,28,29,30], rhythmogenesis, network activity dynamics [31,32,33,34], and neurodegeneration and regeneration [35,36,37].

The combination of electrophysiology and optical methods with microfluidics technology provide a promising approach for isolating the chemical environment of specific regions of the network or individual modules for drug testing and basic physiological research [38,39,40]. Microfluidic devices used for in vitro assembly of neural circuits typically consist of two or more isolated chambers connected by microchannels (see the review by [41]). The ability to locally apply drugs to separate chambers is critical for researching signaling interactions between different neuronal networks or modules. The hydraulic resistance of the microchannels restricts the flow of reagents, enabling the maintenance of different drug solutions [42]. However, selective drug application must also minimize diffusion between the chambers. Diffusion through the microchannels and mechanical disturbances during drug application can lead to instability of network activity and connected neural networks. A microperfusion system has been proposed to address the issue of diffusion through the microchannels [39,40], but this approach requires specialized equipment for continuous active control and can affect cell morphology and network activity [43,44]. Passive microfluidic devices, which are significantly cheaper and easier to use, provide an alternative that can maintain stable neuronal activity during drug treatment procedures in cultured neuronal networks with modular network structures.

We used cultures of hippocampal neurons grown in a two-chamber microfluidic chip combined with a microelectrode array. The shape of the microchannels between the chambers ensured that axons grew predominantly in one direction, forming two neuronal networks with unidirectional synaptic connectivity (the Source and Target networks) [30]. In this study, we present a novel two-layer PDMS chip adapted to standard microelectrode arrays, which allows for the culturing and study of two connected networks of dissociated neurons in isolated environments. To investigate the effect of electrical activity on one network, we applied Tetrodotoxin (TTX) in the Source chamber to suppress the activity of the Source network. The results indicated that stable network activity in the Target chamber was maintained for at least 1–3 h after TTX application, demonstrating the feasibility of local chemical activity modulation and the influence of electrical activity from one network on the other. Our findings reveal that suppression of the Source network’s excitatory synaptic activity resulted in significant reorganization of spontaneous and stimulus-evoked bursting dynamics in the Target network.

## 2. Materials and Methods

### 2.1. Microfluidic Device Fabrication

**Figure 1:**
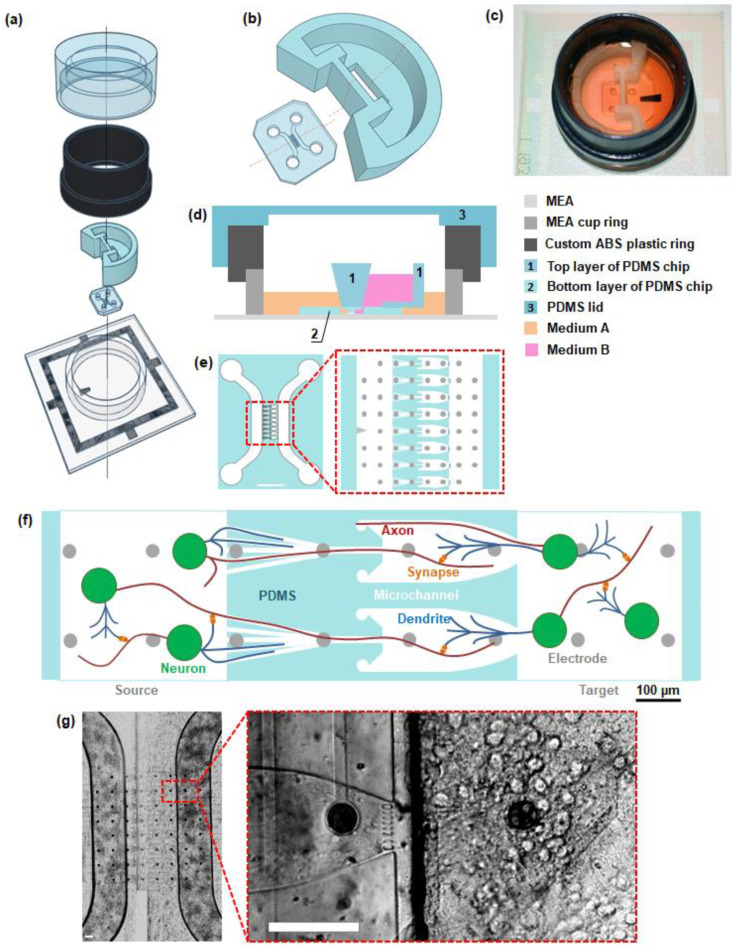
PDMS chip for isolated cultures of neural cells. (**a**) Components and structure of the chip. From bottom to top: microelectrode array, bottom and top layer of the chip, plastic ring, PDMS lid. (**b**) 3D model of the bottom and top layer of the PDMS chip. (**c**) Photo of MEA with the chip without PDMS lid. (**d**) Schematic view of the MEA with the chip (side view along the line from (**b**)). (**e**) Schematic view of the microfluidic chip (scale bar 1 mm) and schematic view of the 8 microchannels of a microfluidic chip connecting two chambers. (**f**) Scheme of unidirectional synaptic connectivity between neural networks, formed by microfluidic chip. (**g**) Light field images of neural cells grown in a microfluidic chip (DIV 15). Scale bar: 100 µm.

Two-layer PDMS chips were fabricated using polydimethylsiloxane (PDMS, Sylgard 184, Dow Corning, Midland, MI, USA) molding technology. A standard soft lithography was used for the bottom PDMS layer fabrication, as described in detail in [30,45,46]. The layer consisted of two chambers (220 µm high) for neuronal cell culture and 8 asymmetric microchannels (5.5 µm high) for neurite outgrowth. Sub-networks in the chambers were designated as Source and Target, according to the predominant direction of axonal growth through the asymmetric microchannels described previously [30] (Figure 1c). We assembled the bottom PDMS layer with MEA consisting of 59 electrodes and one reference electrode (TiN electrodes, 30 µm in diameter, 200 µm in the middle, 1 reference electrode, Multichannel Systems, Reutlingen, Germany). The layer was manually aligned under a binocular with the MEA so that each of the 8 microchannels contained 4 electrodes (Figure 1e). The layer was held in place only by adhesion, so it could be detached after experiments. The MEA dish was formed by a standard plastic ring 6 mm high, ID: 26.2 mm, OD: 30 mm. Additionally, a custom 3D-printed ABS-plastic ring with a height of 12 mm was attached by PDMS (Figure 1a–d). The inner wall of the ABS ring was coated with PDMS.

The top PDMS layer was designed to separate the liquid volumes in both chambers to locally apply reagents to a single chamber (Figure 1a). The shape of the Source chamber in the top PDMS layer was designed to ensure that the reference and recording electrodes were in contact with the same conductive solution. The 3D model of the top layer was designed in Blender software. The mold for the layer was 3D printed from ABS plastic and exposed to acetone vapor for 20 min to smooth out surface irregularities. PDMS was poured into the mold and cured in a dry oven for 6 h at 45 °C. After curing, the PDMS structure was removed from the plastic mold. Then, the top layer was bonded to the bottom layer by gluing with liquid PDMS so that the cavity of the top layer and the Target chamber of the bottom layer formed a single volume (1.1 mL) (Figure 1d). The MEA with the PDMS chip was fixed upside down and placed in a dry oven for 6 h at 60 °C to prevent spilling the liquid PDMS onto the MEA. The culturing surfaces of the MEA-PDMS devices were coated with polyethyleneimine (PEI, 1 mg/mL, Sigma-Aldrich, P3143, Saint Louis, MI, USA) at 4 °C overnight, washed three times with deionized water, and coated with laminin (20 µg/mL, Sigma-Aldrich, L2020, Saint Louis, MI, USA) at 37 °C for 40 min.

A removable PDMS lid was placed on top of the culture dish to prevent evaporation from the medium and fluctuations in osmolarity [47].

### 2.2. Cell Culturing

All experimental procedures were carried out following Protocol #59 (dated 2 December 2022) approved by the Bioethics Committee of the National Research Lobachevsky State University of Nizhny Novgorod (Russia). Hippocampal cells were dissociated from C57BL/6 mouse embryos (E18) and plated in the PDMS chip chambers through seeding wells at an initial density of 6500–7000 cells/mm^2^. After the cells had sedimented and attached to the surface, they were removed from the seeding wells using a P10 pipette tip, leaving only the cells in the chambers. The cells were plated in B-27™ Plus Neuronal Culture System medium (Invitrogen, A3653401, Carlsbad, CA, USA) with 1% glutamine (Invitrogen, 25030-024, Carlsbad, CA, USA), 5% fetal calf serum (Invitrogen, A3160801, Carlsbad, CA, USA), and gentamicin (20 µg/mL) (AppliChem, A1492, Darmstadt, Germany) in an incubator (3552-2, SHEL LAB, Cornelius, OR, USA) at 37 °C, 5% CO_2_, and 95% air covered with PDMS lids to prevent evaporation. The next day, half of the medium was replaced with a medium containing B-27™ Plus Neuronal Culture System, 1% glutamine, 0.4% fetal calf serum, and gentamicin (20 µg/mL). Half of the medium was changed twice a week. For details on cell culture procedures, see [48]. Light field images were acquired with the Axio Observer.A1 microscope (Carl Zeiss, Jena, Germany).

### 2.3. Electrophysiology and Experimental Protocols

A spiking activity was recorded from 59 TiN electrodes of the USB-MEA60-Inv-BC-System (Multichannel Systems, Reutlingen, Germany) at a sample rate of 20 kHz. Detection of the recorded spikes was based on the threshold calculation of the signal median. For details on the spike and the burst detection methods see our previous study [48]. MEA headstages were covered with custom aluminum incubation chambers to maintain stable culture conditions during recording and stimulation. The heating of the headstage and incubation chamber with a microcontroller ensured uniform heating of the air up to 37 ℃ near MEA, preventing condensation. Heated mixture of 5% CO_2_, and 95% air was constantly pumped into the custom incubation chamber.

The general protocol to examine the stability of spiking activity with the local application of reagents included the following steps:

The first 20 min of activity were excluded from the experiment to remove the effect of mechanical impact after the MEA installation in the headstage of the recording system.

1. Low-frequency stimulation applied to 8 electrodes located in the narrow segment of 8 microchannels simultaneously (±800 mV, 260 µs per phase positive first, 200 pulses with 3 s interpulse interval) (LFS 1);
2. One hour of basal spontaneous activity (Spont 1) recorded in culture medium, defined as a basal condition;
3. Repeated low-frequency stimulation (LFS 2);
4. Repeated one hour of basal spontaneous activity recording in the culture medium (Spont 2);
5. Drug application. The drug application was carried out in a laminar at room temperature and atmospheric air. MEA was removed from the headstage. The culture medium was completely removed, and only 3–5 µL remained inside the chip chambers. A volume of 500 µL of the medium was returned to the Target chamber. TTX was added to the 1 mL of culture medium (1 μM) and returned to the Source chamber. In control experiments, this procedure was carried out without TTX application but with similar pipetting of the medium. Then, the MEA was returned to the headstage;
6. Recording of spontaneous activity (20 min). A Medium change and the MEA replacement caused a mechanical perturbation, so we expected temporal instability of the spiking activity during that interval;
7. Low-frequency stimulation (LFS 3);
8. One hour of spontaneous activity recording (Spont 3);
9. Repeat of low-frequency stimulation (LFS 4);
10. Recording of spontaneous activity (one hour) (Spont 4).

Experiments were performed on 75–105 DIV (n = 5 Experiments, 3 cultures).

Protocol for blocking of excitatory synaptic transmission in the Source chamber consisted of following steps:

1. Spontaneous activity recording in culture medium (10 min, Control);
2. Low-frequency stimulation was applied to 8 electrodes located in the narrow segment of 8 microchannels (Control);
3. Drug application. CPP and CNQX solution at a final concentration of 50 μM were added to the Source chamber. The procedure for drug application was the same as for TTX;
4. The first 20 min of spontaneous activity after drug application were excluded from further analysis;
5. Low-frequency stimulation (CPP + CNQX);
6. Spontaneous activity recording (10 min, CPP + CNQX);

Experiments were performed on 21–50 DIV (n = 6 Experiments, 5 cultures).

### 2.4. Analysis of Spiking Activity

All signal analysis and statistics were performed with custom-made software Meaman in MATLAB 2013a (R).

Detection of the recorded spikes was based on the threshold calculation of the signal median. Details of the spike and the burst detection method were described in our previous study [48].

The spontaneous burst characteristics were estimated in the chambers separately. The Spiking Rate (SR) was calculated as a number of spikes per second separately in the Source and Target chambers and normalized to the number of electrodes in the chamber. The probability of the burst propagation between subnetworks in the Source and Target chambers was estimated by previously proposed methods [30].

### 2.5. TTX Application Analysis

SR were normalized for each experiment to the corresponding value of the Basal 1 activity from the first 10 min of the recording.

We compared the SR at the first (Basal 1) and the last (Basal 2) 10 min time intervals of basal spontaneous activity recording and 10 min interval activity in 1st, 2nd, and 3rd hours after manipulation with culture medium and TTX application.

Low-frequency stimulation (LFS) of axons in microchannels evoked a burst of activity in the neural network in the Target chamber. In our study, we considered a network’s response to be eligible for analysis if at least one spike per recording electrode in the chamber was recorded within a time window of 10 to 300 milliseconds after the stimulus. The network response in the Target chamber was evaluated by counting the number of network responses observed during a series of 200 low-frequency stimuli. The resulting values were then normalized with respect to the mean value obtained from the Basal stimulation train.

Statistical analysis was performed with ANOVA analysis and subsequent pairwise comparison was performed by Wilcoxon rank-sum test with Bonferroni correction.

### 2.6. CPP + CNQX Application Analysis

Spiking rate in burst (SR in burst), duration of spontaneous bursts, and interburst intervals were estimated to the Target chamber and normalized to the average value in control recording for statistical comparison. Significant difference among the experimental conditions was assessed by the Mann–Whitney test.

Each stimulus applied to the axons in microchannels could evoke bursts of spikes in both chambers. To characterize the evoked bursts, we estimated the Evoked spiking count as a total number of spikes in the 10–300 ms post-stimulus interval in response to each stimulus for each chamber separately, normalizing it to the number of recording electrodes. For each experiment, the Evoked spiking count values were normalized to the median value estimated in control stimulation train and defined as Evoked Spikes (ES).

### 2.7. Big and Small Bursts Separate Analysis

To separate small and big bursts in spontaneous and evoked activity recordings, we used a K-means clustering algorithm according to the number of spikes per burst in the Target chamber. We estimated two clusters and evaluated cluster separation by calculating the Davies-Bouldin (DB) index [48]. The DB index estimated the ratio between the internal cluster distance and the distance between clusters. Small values for the DB index corresponded to compact clusters whose centers were located far from each other. The recordings, in which DB values ranged from 0 to 0.65 indicated “robust” clustering, and the small and big bursts were analyzed separately.

According to clusterization analysis, we identified “big” bursts of spontaneous activity and “big” responses in evoked activity.

Furthermore, the bursts in the Target network caused by the Source network’s activity were designated as Received bursts. The rest of the big bursts in the Target network were called Intrinsic bursts.

For each big response, we estimated a Post-stimulus time histogram (PSTH) for each chamber separately as a number of spikes in each 10-ms time interval (spikes per bin). We estimated the delay of the maximum number of spikes in PSTH (Max delay) and ES of big responses in the Target chamber before and after CPP + CNQX mixture application. Statistical analysis was performed by the Mann–Whitney test. We considered *p* < 0.05 as a significant difference. All signal analysis and statistics were performed with custom-made software in MATLAB (R).

### 2.8. Functional Connectivity

To estimate functional changes of the Target network after suppression of input activity in the Source network, we analyzed spike propagation characteristics during the burst generation between the electrodes. Conditional firing probabilities (CFP) were used to determine functional connectivity in the recordings of spontaneous activity [22]. CFPs were calculated for all possible pairs of electrodes in the Target as the probability to record a spike at electrode j at t = τ (300 ms > τ > 0), given that one was recorded at electrode i. Two electrodes were defined as functionally connected if a CFP_ij_ curve was not flat. Functional connections between electrodes i and j were characterized by strength S_ij_^strength^ (ranging from 0 to 1) and latency S_ij_^latency^ (ms).

To measure the difference between the connectivity formed by the bursts in two recordings, we estimated a Euclidean Distance of two connectivity matrices CFP_ij_ [24]:

(1) $${\mathrm{ED}}^{\mathrm{strength}}\left(R_{1},R_{2}\right)=\sqrt{\overset{n}{\underset{i=1}{\sum }}\overset{n}{\underset{j=1}{\sum }}{\left[S_{\mathrm{ij}}^{\mathrm{strength}}\left(R_{2}\right)-S_{\mathrm{ij}}^{\mathrm{strength}}\left(R_{1}\right)\right]}^{2}}$$

(2) $${\mathrm{ED}}^{\mathrm{latency}}\left(R_{1},R_{2}\right)=\sqrt{\overset{n}{\underset{i=1}{\sum }}\overset{n}{\underset{j=1}{\sum }}{\left[S_{\mathrm{ij}}^{\mathrm{latency}}\left(R_{2}\right)-S_{\mathrm{ij}}^{\mathrm{latency}}\left(R_{1}\right)\right]}^{2}}$$

where R_1_ and R_2_—recordings 1 and 2; n—number of electrodes in the Target chamber.

CFPs were calculated in the Target chamber under Control and after CPP + CNQX application to the Source chamber conditions. In Control condition, we excluded the bursts which were spontaneously evoked from the Source network, leaving only Intrinsic big bursts for connectivity analysis. The recording after CPP + CNQX treatment to the Source network was divided into two 5 min recordings (CPP + CNQX 1 and CPP + CNQX 2), from which the big bursts were used for CFP estimation. Then, using ED we compared the connectivity of the Intrinsic bursts in Control with the connectivity in CPP + CNQX 1 (ED^strength^ (Intrinsic, CPP + CNQX 1) and ED^latency^ (Intrinsic, CPP + CNQX 1)). To evaluate if connectivity changes appeared due to a suppression of input activity we estimated connectivity changes with ED between the bursts in CPP + CNQX 1 and CPP + CNQX 2 (ED^strength^ (CPP + CNQX 1, CPP + CNQX 2) and ED^latency^ (CPP + CNQX 1, CPP + CNQX 2)). ED^strength^ and ED^latency^ were compared in two conditions separately. Statistical analysis was performed by the Wilcoxon rank-sum test.

### 2.9. Evoked Burst Profiles

To represent a dynamics of spiking activity in stimulus response for each recording electrode in the Source and Target chambers, a diagram of spiking profile was constructed displaying the number of evoked spikes for each 2 ms bin from 10 to 300 ms after the stimulus and averaged across all stimuli. The average number of spikes were represented in color.

### 2.10. Selective Network Dynamics

We estimated the number of Evoked spiking counts occurring between 10 and 300 ms after stimulus, as well as the First spike timing following 10 ms for each recording electrode in the Target chamber during low-frequency stimulation (200 stimuli, 3 s interstimulus interval, 8 electrodes in microchannels). Subsequently, the Evoked spiking count and First spike timing values were compared for each electrode before and after the application of CPP + CNQX to the Source chamber. Recording electrodes were defined as selective if there was a significant difference between the Evoked spiking count and First spike timing values during low-frequency stimulations under Control and CPP + CNQX conditions (Mann–Whitney Rank Sum Test, *p* < 0.05). The number of selective electrodes was expressed as a percentage of the total number of active electrodes in the Target chamber. Statistical analysis was performed by the Wilcoxon rank-sum test.

## 3. Results

Microfluidic chips for co-culturing several neural networks typically have a height of several millimeters, limiting the maximum volume of medium in each chamber for separate chemical manipulation. To address this, we developed a two-layer PDMS structure that extends the volumes of separate chambers connected only by microchannels filled with neurites (Figure 1). The bottom layer was produced using a common method of photolithography, and the top layer was made using widely used 3D printing methods (see Section 2). The bottom layer provides unidirectional synaptic connections from the network in the Source chamber to the Target chamber. The top layer increases the volume of the chambers, enabling investigation of the interaction between sub-networks by applying blocking agents to the Source chamber. Using the 3D model of our platform, we estimated that the hydrostatic equilibrium between the two chambers was established when adding approximately 1400 µL into the Source chamber and 500 µL into the Target chamber. In our experiments 1000 µL and 500 µL of medium solution were added to the Source and Target chambers, respectively, to create hydrostatic pressure and prevent the diffusion of chemicals from the Source chamber. Neglecting the capillary pressure, such liquid level difference provided the pressure about 17 Pa. According to the Hagen–Poiseuille equation, the flow rate in the channels was ~ 13 pl/s, which corresponded to the average velocity 15–150 µm/s, depending on the channels’ cross-section. This velocity is high enough to prevent diffusion from the Source chamber to the Target chamber. Taking into account the capillary pressure in the chambers, the backflow might be slightly larger, which does not lead to significant changes in the estimations.

Next, we separately seeded dissociated hippocampal neurons in each chamber and cultured them for several months.

### 3.1. Spiking Activity Dynamics under Conditions of Local Application of TTX

First, we confirmed that asymmetric microchannels formed unidirectional spiking activity propagation between networks. The propagation probability of spontaneous bursts from the Source to Target chamber was 25.9 ± 19.3% and only 0.7 ± 0.96% in the opposite direction before TTX application (Basal 2, n = 5 experiments, 3 cultures, mean ± s.d.). Then, we examined the chip’s ability to maintain stable activity in the Target chamber while TTX was applied in the Source chamber. We analyzed the spontaneous and stimulus-evoked activity of the network in the Target chamber before and after TTX treatment in the Source chamber. The spiking rate (SR) was stable during two hours of basal spontaneous activity recording in both networks in the Source and Target chambers, verifying the stability of the experimental conditions.

**Figure 2:**
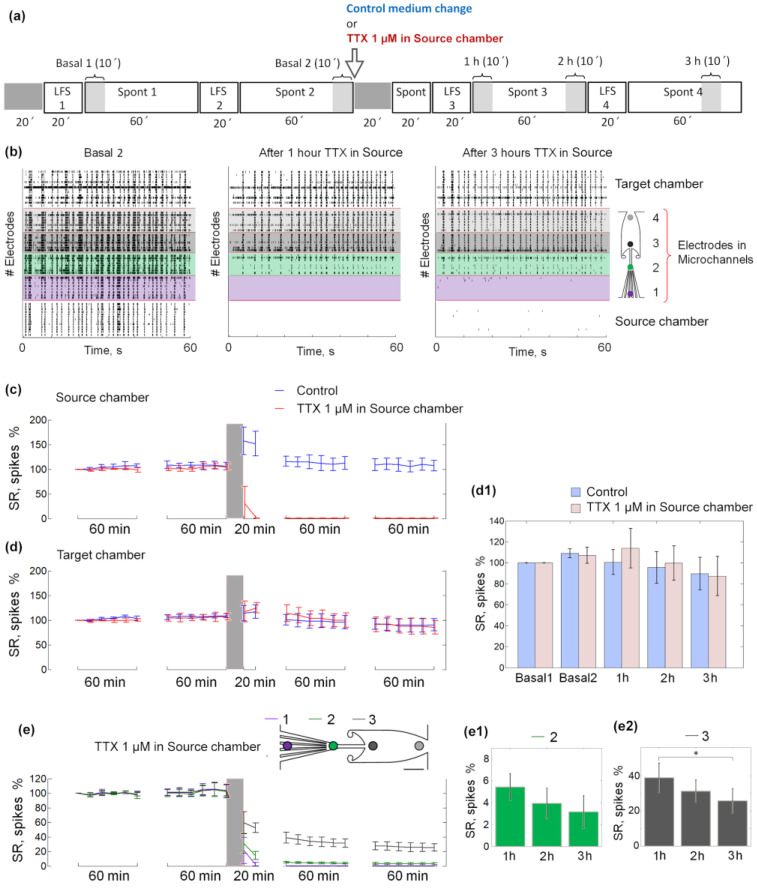
Network activity with 1μM TTX application in the Source chamber. (**a**) The experimental protocol consisted of spontaneous activity recording (Spont) and low-frequency stimulation (LFS) series. The arrow indicates the application of TTX to the Source chamber or the change of medium in control experiments. (**b**) An example of raster plots showing 60 s of spontaneous activity in basal conditions (**left**), 1 h (**middle**), and 3 h (**right**) after TTX application in the Source chamber. Each dot represents a spike, each row an electrode. The electrodes are highlighted in color according to the scheme on the right. (**c**,**d**) Spiking rate (SR) of spontaneous network activity normalized to the SR in the first 10 min of the recording. Each dot represents SR (Mean ± s.d.) during 10 min for the neuronal network in the Source chamber (C) and the neuronal network in Target chamber (**d**). The red line corresponds to the activity of cultures with 1μM TTX in the Source chamber, blue line corresponds to the activity of control cultures. The gray-shaded area indicates a 15–20 min recording stop during and after manipulation with culture medium and TTX treatment. (**d1**) Diagram of SR at the first (Basal 1) and the last (Basal 2) 10 min time intervals of basal activity recording, and 1, 2, and 3 h after manipulation with culture medium and TTX application (1–3 h, respectively) in control experiments (blue), and with 1 μM TTX in the Source chamber (red). (**e**) SR of spontaneous network activity normalized to the SR in the first 10 min of the recording. Each dot represents SR during the 10 min for the neuronal network in electrodes nearest to the Source chamber (marked as 1, purple line), electrodes in the narrow section of microchannels (marked as 2, green line), and electrodes in the wide section of microchannels (marked as 3, gray line) of each culture with 1μM TTX in the Source chamber. (**e1**,**e2**) Diagram of SR at 10-min time intervals of spontaneous activity 1, 2, and 3 h after TTX application into the Source chamber (1–3 h, respectively), recorded by the electrodes in the narrow section of microchannels (**e1**) and 8 electrodes in the wide section of microchannels (**e2**). Data are represented as Mean ± s.d. Statistical analysis was carried out using ANOVA and a Wilcoxon rank-sum comparison test. Five experiments were in control and TTX groups, * *p* < 0.05.

The SR of the Source network decreased to zero during the first 20 min after TTX application. At the same time, the SR of the Target network did not change for 3 h (Figure 2c,d,d1) as in control experiments. Note that there was a slight trend towards a decrease in SR in the Target network, both in the control experiments and with TTX treatment.

Two series of low-frequency stimuli with an interval of 1 h were applied to assess the stability of evoked activity (network response) in the Target chamber under basal conditions (LFS 1 and LFS 2). The network response was normalized to the mean value during LFS1. After 1 h, the response did not change and was equal to 93 ± 26.4% during LFS2 (Wilcoxon rank-sum test with Bonferroni correction, *p* = 0.6825).

Then, the low-frequency stimuli were applied with a 1-h interval after the application of TTX. The responses to LFS 3 and LFS 4 in the Target network were 10.2 ± 10.8% and 9.8 ± 16.3%, respectively, and were significantly lower than the responses to LFS 1 and LFS 2 (Wilcoxon rank-sum test with Bonferroni correction, *p* < 0.05).

Although the activity in the Source chamber was blocked, the axons in the microchannels were not completely inactive; electrical stimuli still evoked action potentials transmitted along axons, resulting in a response in the Target chamber.

The application of TTX was expected to diffuse in the microchannels and affect spiking activity on the electrodes. Thus, we analyzed the activity separately on electrodes located in the region nearest to the Source chamber (1st, purple), the narrow section (2nd, green, used for stimulation), and the wide section (3rd, gray). In the narrow section (2nd electrode), activity decreased to 3–5% and in the wide section (3rd electrode) it decreased to 30–40% compared to baseline activity. Despite a slight downward trend after TTX application, no significant differences in SR in the microchannel regions were observed between the activity at 3 h (Figure 2e1,e2)in the narrow section (2nd electrode). The SR in the wide section (3rd electrode) was lower 3 h after TTX application compared to 1 h after (Wilcoxon rank-sum test with Bonferroni correction, *p* < 0.05).

### 3.2. Network Activity in the Target Chamber under Suppression of Excitatory Synaptic Transmission in the Source Chamber

We estimated the spontaneous activity propagation from the Source to Target chamber through unidirectionally formed connections in the microchannels in Control conditions. The propagation probability from the Source to Target chamber was 25 ± 14% before CPP + CNQX application (n = 6 experiments, 5 cultures, mean ± standard deviation). The opposite direction from the Target to Source chamber was 3 ± 5%, confirming the unidirectional connection between the networks.

**Figure 3:**
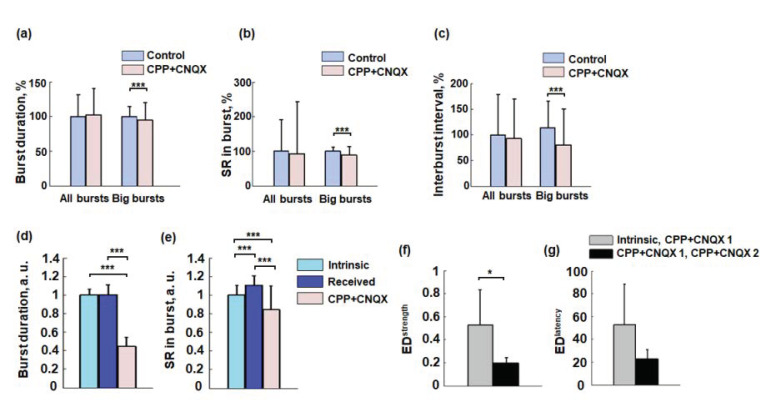
Spontaneous activity before and after suppression of excitatory synaptic activity in the Source chamber. (**a**–**c**) All spontaneous bursts and big spontaneous bursts duration, spiking rate in burst, and interburst interval in the Target chamber (median ± m.a.d., n = 6 experiments, Mann–Whitney test, *** *p* < 0.001). (**d**,**e**) Duration and spiking rate of the Intrinsic, Received big bursts under Control condition and big bursts under CPP + CNQX condition (see Methods) (median ± m.a.d., n = 6 experiments, Mann–Whitney test with Bonferroni correction, *** *p* < 0.001). (**f**,**g**) Functional connectivity changes measured by Euclidean distance between connectivity strengths (ED^strength^) and connectivity latencies (ED^latency^) (see Section 2). Connectivity was compared for two sets of the big bursts (Intrinsic, CPP + CNQX 1) and (CPP + CNQX 1, CPP + CNQX 2) (median ± m.a.d., n = 6, Wilcoxon rank-sum test, * *p* < 0.05).

To study functional inter-network interactions, we blocked excitatory synaptic transmission in the Source network and analyzed changes in spontaneous and stimulus-induced network activity in the Target network. Characteristics of network activity such as burst durations, spiking rate in bursts, and interburst intervals were estimated for spontaneous network activity in the Target chamber and normalized to the median value in the basal condition. The distribution of spontaneous burst characteristics often included two clusters (big and small bursts) which were separated using the K-means algorithm. We found no significant differences between all (big and small) spontaneous bursts in the Target chamber before and after suppressing synaptic transmission in the Source chamber. However, the durations of the big bursts significantly decreased from 100 ± 140% to 94 ± 25%, the spiking rate significantly decreased from 100 ± 11% to 88 ± 23%, and the interburst interval significantly decreased from 100 ± 45% to 70 ± 60% (median ± m.a.d., Mann–Whitney test, *p* < 0.001) (Figure 3a–c). Therefore, the lack of influence from the Source chamber activity resulted in a decrease in burst duration in the Target chamber with fewer spikes and more frequent bursts.

Then, we analyzed the bursting activity changes in more detail. In Control condition, the activity consisted of the big bursts evoked from the Source network and Intrinsic big bursts generated by the neurons in the Target network. We hypothesized that observed changes of the bursting activity were caused by CPP + CNQX suppression of propagated bursts and Intrinsic bursts would be similar to the bursts after CPP + CNQX application. We found no difference for the burst duration between the Intrinsic and the Received bursts (Figure 3d), but after CPP + CNQX it was significantly reduced (Mann–Whitney test, *p* < 0.05). The SR of the Received bursts was significantly higher than Intrinsic bursts in control conditions (Figure 3e) but after CPP + CNQX the SR significantly reduced (Mann–Whitney test, *p* < 0.05).

We estimated functional connectivity characteristics between electrodes in the Target network during the formation of the big bursts (see Section 2). We hypothesized that the Intrinsic bursts in Control condition would involve the same functional connections for burst generation as any bursts after CPP + CNQX application to the Source network. We compared connectivity matrices of the Intrinsic bursts in Control condition with the bursts in the first half of the recording after CPP + CNQX (Intrinsic and CPP + CNQX 1 condition). Such changes were then compared with the changes within the CPP + CNQX application (CPP + CNQX 1, CPP + CNQX 2). Comparison by simple Euclidean distance measure revealed that the connection strength significantly changed after suppression of the Source activity, indicating functional reorganization (Figure 3f, n = 6 experiments, 5 cultures; Wilcoxon rank-sum test, *p* < 0.05). The latency of the connections did not change significantly (Figure 3g, n = 6 experiments, 5 cultures; Wilcoxon rank-sum test, *p* = 0.063).

**Figure 4:**
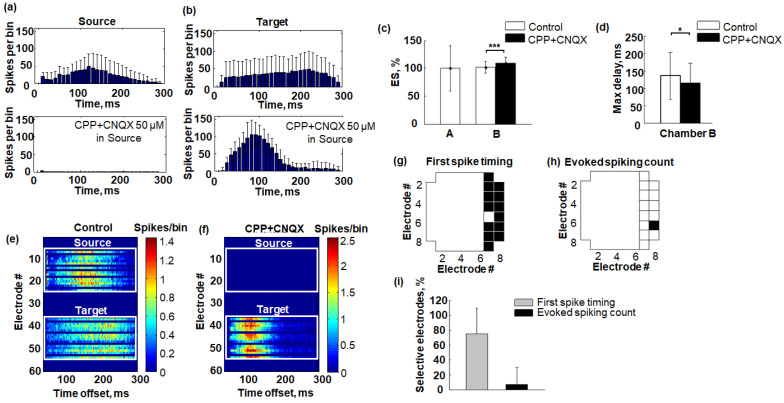
Response to stimulation of axons in microchannels before and after blocking synaptic transmission in the Source chamber. (**a**) An example of network response post-stimulus time histogram (PSTH) in the Source chamber under basal and under CPP + CNQX conditions. (**b**) An example of network response PSTH in the Target chamber under basal conditions and after the application of the mixture of CPP + CNQX to the Source chamber. (**c**) The evoked spikes (ES) are normalized to the median value under basal conditions (median ± m.a.d., n = 6 experiments, Mann–Whitney test, *** *p* < 0.001). (**d**) The delay of the PSTH maximum (Max delay) in the Target chamber (median ± m.a.d., n = 6 experiments, Mann–Whitney test, * *p* < 0.05). (**e**,**f**) An example of the spiking profile of network response for each recording electrode in the Source and Target chambers under basal conditions (**e**) and after the application of the mixture of CPP + CNQX to the Source chamber (**f**). (**g**,**h**) An example of selective electrodes in the Target chamber comparing First spike timing (**g**) and Evoked spiking count (**h**) in network responses before and after application of CPP + CNQX to the Source chamber. (**i**) Number of selective electrodes in the Target chamber comparing First spike timing (**c**) and Evoked spiking count (median ± m.a.d., n = 6, Wilcoxon rank-sum test, *p* = 0.063).

Then, we estimated the response induced by low-frequency stimulation of the axons in the microchannels (Figure 4a,b). The number of evoked spikes (ES) was evaluated in both chambers and then normalized to the mean value under basal conditions. As expected, the spiking activity in response to stimulation in the Source chamber completely disappeared. However, ES in the Target chamber increased from 100 ± 10% to 108 ± 10% (median ± m.a.d., Mann–Whitney test, *p* < 0.001) (Figure 4c).

The dynamics of the burst response in the Target chamber significantly changed, comparing control conditions and excitatory synaptic activity suppression in the Source chamber. Specifically, the number of evoked spikes increased faster during burst generation. We estimated the delay of the PSTH maximum (Max delay) for big responses in the Target chamber and found that the Max delay significantly decreased from 136 ± 64 ms to 115 ± 55 ms (median ± m.a.d., Mann–Whitney test, *p* < 0.05) (Figure 4d).

Then, we analyzed changes of spiking activity in more detail. We estimated the spiking rate profiles of the responses for all electrodes separately (See Section 2, Figure 4e,f). Note that in the presented experiment, the spiking activity in the Target was uniformly distributed at 300 ms of post-stimulus period and after the Source network suppression it changed and appeared mostly at 0–150 ms interval. We characterized the response characteristic in each electrode with a timing of the first spike and evoked spiking count (See Section 2). We then estimated a statistical difference for that characteristic by comparing it in two conditions for each electrode. The electrode in which the characteristic significantly changed after suppression of the Source activity demonstrated selective dynamics in response to various input spiking patterns evoked through the microchannels, i.e., was defined as a selective electrode. In one example experiment, we found 13 selective electrodes (out of 14) measuring First spike timing (Figure 4g) in response and only 1 (out of 14) measuring Evoked spiking count (Figure 4h). On average, the timing characteristic provided a higher number of selective electrodes (75.1 ± 34.4% electrodes, median ± m.a.d., n = 6) compared to spiking count, i.e., spiking frequency (7.1 ± 22.2% electrodes, median ± m.a.d., n = 6). However, a statistical significance was not observed (*p* = 0.1126, Mann–Whitney test, n = 6) (Figure 4i).

## 4. Discussion

Here we present a novel PDMS structure adapted for use with microelectrode arrays, which enables the cultivation of two interconnected neural networks in isolated environments. The design of the device is based on the well-established concept of separate chambers with partially isolated cultured networks connected by axons in microchannels. We irreversibly bonded the top layer to the bottom layer of the PDMS chip to increase the volume of the medium in each chamber, allowing for selective drug treatment and analysis of inter-network interactions on a time scale of several hours without the need for an additional microperfusion system to control diffusion between the chambers. The ratios of plated cell number to media volume were 16 cells/µL and 32 cells/µL in the Source and Target chamber, respectively, which was comparable with standard culture protocols (20–50 cells/µL, [49]; about 120 cells/µL, [50]). It should be noted that in our study, the PDMS structure was reversibly bonded to the microelectrode array, allowing for multiple uses. However, this attachment is relatively weak for active perfusion studies involving high pressure. In such cases, the PDMS chip can be filled with iron micro-powder and attached to the microelectrode array using neodymium magnets [40].

We applied TTX to a single chamber, which suppressed the network’s spiking activity, while the activity in the second chamber did not significantly change over 3 h (Figure 2). A decrease in SR in the wide section of the microchannels was observed between 1 and 3 h, but not the first two hours after TTX application. This decrease could be caused by TTX diffusion, which could be detected in a limited volume of the microchannel but did not significantly change the activity of the neural network in the Target chamber. The asymmetric geometry of the microchannels guided axons to grow dominantly in one direction [30], providing burst propagation from Source to Target chamber. In our previous study, when we introduced similar asymmetric microchannels between two neuronal networks, we visualized unidirectional connectivity with b3-tubulin, tau protein, and MAP2 (microtubule-associated protein 2) [30,51]. The spiking rate in the Target chamber did not change in the absence of input signals from the Source chamber due to TTX or CPP + CNQX application, indicating that it was largely determined by the independent dynamics of the single network. Previous studies reported significant changes in the spiking activity of synaptically connected neural networks when interaction was disrupted [52,53,54]. Laser lesions of the axons induced a significant decrease in the network’s mean firing rate [53,54] and complete recovery occurred 2 h after the lesion [53]. Note that such a decrease was not observed in our approach. Also note, we selectively blocked network activity in isolated chambers of microfluidic chip with conventional chemical methods, allowing reversible suppression of input signals from the associated network without damaging the cells, compared to previous studies.

Recently, a method to suppress neural activity by delivering heat to neurons using thermoplasmonic gold nanoparticles has been proposed [55,56]. A decrease of 50% or more in the overall spiking rate was observed in the associated module when the activity of another module was suppressed by this approach, without injuring the axons forming intramodular connectivity [56]. However, in our experiments, such drastic changes in the spiking activity of connected networks were not observed, which may be due to the higher cell density in the culture, and thus, could provide a large number of connections within a single network that provides stable activity dynamics.

We found no significant differences between burst characteristics (SR in bursts, burst duration, interburst intervals) in the Target chamber before and after excitatory synaptic transmission suppression in the Source chamber by CPP + CNQX considering all spontaneous bursts. In previous studies, networks were physically sectioned using a UV laser [52], and this led to no change in burst frequency in isolated areas from what it was before sectioning. Additionally, the laser ablation of intermodular axons did not change the bursting rate in isolated modules [54]. This result agrees with the findings of no changes in interburst intervals of all bursts in our study.

The activity of mature neuronal cultures with high cellular density often involves two well-distinguished types of the bursts, high and low, based on its spiking rate and duration [57]. We suggest that such big bursts involved most of the neurons in the network which represented stable network dynamics and were analyzed in more detail. We observed a significant decrease in the duration and spiking rate of big bursts, as well as a decrease in interburst intervals in the spontaneous activity in the Target chamber after CPP + CNQX application in the Source chamber (Figure 3). We also separately analyzed two types of the big bursts in the Target network: Intrinsic bursts that were generated by independent dynamics of the single Target network and Received bursts that were generated in response to input spiking activity from the Source via microchannels. We hypothesized that Intrinsic big bursts would not significantly change in terms of integral characteristics (burst duration and spiking rates) and inter-electrode functional relation analyzing functional connectivity after suppression of input activity from the Source. The experiments were performed on mature cultures in which synaptic development is thought to be stabilized after DIV 20 [58]. Surprisingly, we found that big bursts in the Target significantly changed, indicating functional reorganization of synaptic connectivity. We suggest that balance between activity and connectivity was disturbed with Received bursts abolishment and new balance appeared producing new functional state of the network [22,24].

The spiking rate of the response in the Target to low-frequency stimulation applied to the microchannels increased after CPP + CNQX was added to the Source. The delay of network-bursting activity in response to the stimulus in Target chamber also decreased significantly (Figure 3), possibly due to increased excitability and reduced refractoriness following spontaneous bursts from the network in the Source chamber via axons in the microchannels. Electrical stimulation of the microchannels could induce retrograde spiking activity back to the axon soma in the Source chamber, which then could induce the bursting activity through the microchannels and evoke the resulting bursting activity in the Target. However, the stimulation of the microchannels in the absence of synaptic activity from the Source chamber caused by CPP + CNQX application also induced responses in the Target chamber only by direct activation of presynaptic axons in the microchannels, and hence directly activated the network in the Target chamber. We also suggest that altered input patterns induce various responses in the Target chamber. Previously, it was shown that precise timing of initial spiking activity of the bursts and the spiking rate in response to electrical stimulus can be used to encode information about the spatial location of the stimulation source in the network [48,59]. Selective spiking activity to various input signals distributed in the network with various magnitudes in different neurons (electrodes of the MEA) represents information processing features of the network. The spiking activity of certain neurons at the beginning of a burst determined the dynamics of the subsequent activity [60,61]. We found similar selective dynamics in the Target network comparing the stimulus responses in two conditions. Higher percentage of electrodes responded selectively to the stimulus according to the first spiking times of the bursting response compared to the spiking rate of the response (Figure 4f). However, a significant difference of such temporal and frequency features were not observed, presumably due to insufficient experiment repetitions, which can be addressed in further studies.

Overall results suggest that to study synaptic transmission through a group of unidirectional synaptic connections on the network level in such an in vitro model of unidirectionally connected neural network it is necessary to suppress the Source network activity to prevent the effects of retrograde activity distortion.

Three-chamber microfluidic chips with a synaptic Target chamber between cell compartments were used to study synaptic transmission between subnetworks [8]. In contrast to these works, our aim was to selectively affect one of the networks, leaving synaptic contacts unaffected, thus preserving the ability for registration and stimulation of input signals to uncover detailed characteristics of the interaction between the networks.

In contrast to previous studies, our aim was to selectively target one of the networks while leaving the synaptic connections intact, thus enabling us to record and stimulate input signals and uncover the detailed characteristics of interaction between the networks. To the best of our knowledge, this methodology and the results of inter-network interaction with suppression were presented here for the first time.

In conclusion, we evaluated the efficacy of our developed device by suppressing the activity of one network and found that stable experimental conditions could be maintained for several hours. Moreover, we utilized the device to examine the modifications in neuronal network activity due to intermodular or exogenous factors. This study highlights the potential applications of our developed device for investigating neural network interactions in basic Neuroscience and drug screening.

## Data Availability

The data presented in this study are available on request from the corresponding author.
